# Representationalism can connect neuroscience and philosophy

**DOI:** 10.3389/fcogn.2025.1623712

**Published:** 2026-01-02

**Authors:** Albert Kok

**Affiliations:** Psychology Department, Brain and Cognition Group, University of Amsterdam, Amsterdam, Netherlands

**Keywords:** dualism, philosophy of mind, cognitive neuroscience, theory of memory-representations, consciousness, intentionality, emergence, qualia

## Abstract

Representationalism is the theory that our conscious perception of the world is mediated by mental representations, rather than being a direct encounter with reality. In this article, we define representations in terms of a unified theory of long-term memory that incorporates both its explicit and implicit divisions. Apart from these integrative features, the theory offers the possibility for reconciling perspectives in neuroscience and the philosophy of mind. We address various areas of debate, focusing on concepts such as consciousness, intentionality, emergence, and qualia. We draw the following conclusions based on our framework applied to representational systems. First, conscious experience results from the retrieval of the contents of explicit memory representations from short-term memory. Regarding intentionality, the strong link between intentionality and memory representations allows us to define intentionality, capturing both the “what is it” and “what it is like” aspects. Finally, notions referring to the subjective experiential content of consciousness, such as emergence and “qualia”, are integral to all conscious experience, reflecting memory-emotion interactions realized in neuro-affective networks. Ultimately, we conclude that concepts from the philosophy of mind can be harmonized, in a non-reductionist way, with neurocognitive theories that define memory representations as multilevel networks of large-scale brain systems.

## 1 Introduction

The traditional approach in neuroscience was to study brain structures without considering the mind, bypassing the brain's crucial role as a central integrator. The famous quote by Marvin Minsky, “the mind is what the brain does” (Minsky, 1985), could have tempted some to suggest that brain scans may suffice to reveal the nature of mental states, and even the content of our thoughts. This tendency toward “neuro-simplification” is probably most evident in neuroscientists who traditionally analyzed the brain's structure at the microlevel of neurons.

The study of the brain, aimed at unraveling the nature of mental processes, clearly needs a convergent, more macro-oriented research ideology, such as cognitive neuroscience. Its mission was to combine paradigms from cognitive psychology with methods from neuroscience to study brain structure and functions. Functional connectivity, in particular, has become the gold standard for describing the temporal dependencies among neuronal activation patterns across anatomically separated brain regions (Sporns et al., 2005, 2014). Human neuroimaging research has further transitioned from mapping local effects to developing predictive models of mental events that integrate information distributed across multiple brain systems (Kragel et al., 2018). Despite earlier methodological limitations, the field is developing rapidly, incorporating new advanced techniques at both the macro- and micro-levels (Bassett and Sporns, 2017; Ross and Bassett, 2024, for recent reviews).

Philosophy is an a priori discipline devoted to theorizing and thinking, freed from the tedious job of collecting data sets in the lab or the field. Dennett said this was not always favorable, “since one can make philosophy out of just about anything” (Dennet, 2006). To a certain extent, this also holds for philosophers of mind who struggle with the theories and constraints imposed by neuroscience. Some of its definitions, such as “philosophy of mind is the study of mindful things just insofar as they are minded” (Lowe, 2000, 2008), may not be directly transparent. Another, more common definition emphasizes its commitment to a subjective experiential approach more directly: that philosophy of mind is concerned explicitly with general questions about the nature of mental phenomena, such as feeling, perception, consciousness, and sensory experience, thus contributing to bridge the explanatory gap between the physical brain and subjective experiences (Quine, 1975; Dempsey, 2004; Dennett, 2005; Churchland, 2002; Perring, 2011; Mendelovici and Bourget, 2020).

**Figure 1:**
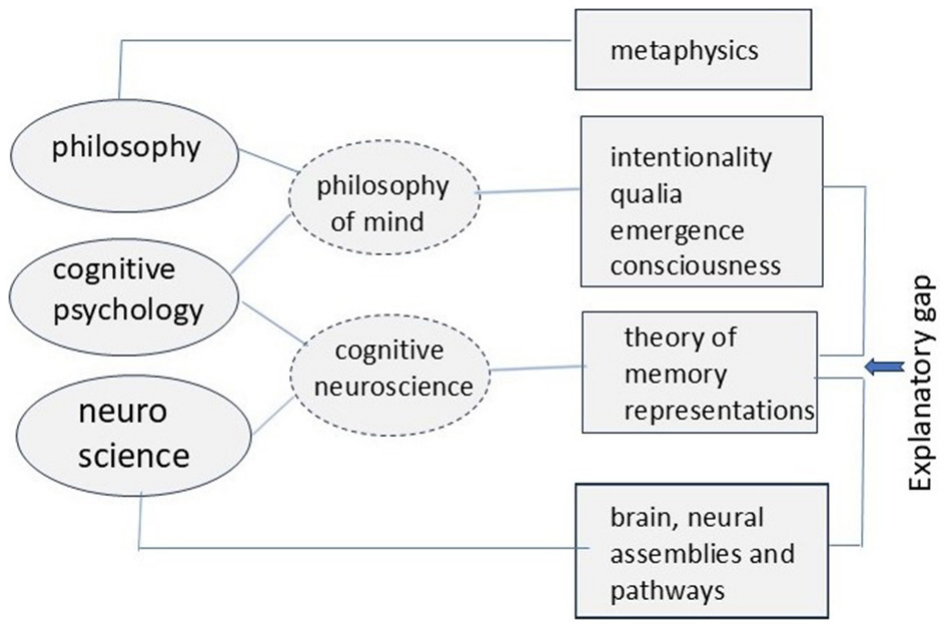
Outline of central disciplines and concepts of the present article. Left: the three involved disciplines and two subdisciplines (dotted lines). On the right, we present the key concepts and functions discussed from the perspectives of philosophy of mind and cognitive neuroscience. According to our theory of memory representations, further elaborated below, cognitive neuroscience bridges the explanatory gap between concepts in philosophy of mind (see arrow) and those in neuroscience.

This article aims to bridge the explanatory gap between philosophical concepts and neuroscience, using a memory model derived from cognitive neuroscience (Figure 1). Given its close links to cognitive psychology and neuroscience, we propose that cognitive neuroscience is well-positioned to address this problem. In the following sections, we will provide a brief overview of the historical background of mind-body dualism. We then introduce a theoretical model of memory representations that will serve as a reference point for discussing concepts such as consciousness, intentionality, the “hard problem”, emergence, and qualia in the following sections of this article (see Box 1 for a glossary of terms used in this paper).

Box 1 **General glossary of terms.**

- *Dualism*: the theory that mind and brain are different and separate phenomena.
- *Emergence*: In philosophy, emergence often means irreducibility, implying that although mental properties arise from physical properties of the brain and supervene on them, they can never be reduced to them. Alternatively defined in neuroscience as: multi-level representations, which, emergently and in parallel, give rise to multimodal, ‘spatially wide super inferences corresponding to phenomenal experiences' (Pennartz, 2022).
- *Explanatory gap:* a term introduced by philosopher Joseph Levine in his 1983 paper, where he used the sentence, “pain is the firing of C-fibers”, to clarify that while it might be valid in a physiological sense, it does not help us to understand how pain feels.
- *Hard problem*: a term coined by philosopher David Chalmers, implying that no neural or behavioural explanation could explain the character of a subjective experience, not even in principle.
- *Idealism*: a movement in philosophy claiming that reality is entirely a mental construct, and that ideas are the highest type of reality or have the most substantial claim to being considered “real”.
- *Intentionality*: the ability of minds and mental states to be about, to represent, or to stand for things, properties, and states of affairs. Divided into objective (“what it is”) and subjective (“what it is like”) intentionality.
- *Materialism:* the theory that the mind and brain have the same physical substrate. Also referred to as determinism, physicalism, or reductionism.
- *Multimodal network:* a network that integrates various sensory (visual, somatosensory, auditory) modalities
- *Multilevel network:* a network that comprises various levels or hierarchical organization: neurons, modules, and large-scale networks.
- *Naturalism:* the principle that scientific methods should also be used in philosophy, and that science and philosophy are not always distinct from one another, but instead form a continuum
- *Phenomenology*: a philosophical movement opposite to idealism, stating that any phenomenological description is to be performed from a first-person point of view, to ensure that the respective object is described exactly as it is perceived from the sensory data.
- *Qualia:* The subjective quality (alias phenomenal experience) of perceptions, represented in the brain as information structures that have a physical as well as a phenomenal meaning.
- *Representation:* refers to a hypothetical internal cognitive symbol that represents external reality. In the present paper, interpreted in terms of memory representations, created by correlated transient activity, both electrical and chemical, in a population or assembly of neurons in several cortical areas.
- *Representationalism*: is the theory that our conscious perception of the world is mediated by mental representations, rather than being a direct experience of reality. In an operational sense, it refers to the brain's cognitive system's capacity to represent, store, and retrieve information.

## 2 Metaphysics, dualism, materialism

The metaphysical orientation of philosophy initially overlapped with theology, engaging with the divine and the transcendent in questions about the 'mystery of the human soul', free will, and the meaning of human existence (Schumacher, 2022). The theistic orientation also formed the basis of dualism: the view that the mind (or soul) and the material brain are strictly separate. The French philosopher and mathematician René Descartes became the embodiment of dualism in the 17th century. Although dualism is subdivided into different versions (Lowe, 2008; Pecere, 2020), we shall here deal with the substantial version in which Descartes proposed a strict separation between the material world (*res extensa*) and mind (*res cogitans*). A radically different materialistic—or deterministic—view in the history of the philosophy of mind was introduced during the Enlightenment in the 18^th^ century by the French physician and philosopher Julien Offray de La Mettrie. Materialism, the counterpart of dualism, posits that all things, including the human body, mind, and consciousness, are the result of material interactions between material things. In his essay “Man as a Machine” (*L'Homme machine*), La Mettrie declared that “man is nothing more than a perceiving, thinking, and feeling body” (Wolfe, 2020). Perhaps more complicated than an animal, but not essentially different.

Brain science gradually abandoned the notion of the soul's or mind's immaterial nature in the following centuries. This was exemplified by Pierre Cabanis in 1824 (see Turgeon and Whitaker, 2000), who stated that “the brain secretes thought, like the liver secretes bile”. Further developments in the 19th century, notably Darwin's revolutionary theory of natural selection, laid the groundwork for more biologically oriented theories of the human mind-body relationship.

Is dualism outdated? From a cognitive neuroscience perspective, the intuitive answer would be “yes”. Our mind is not a metaphysical or non-material thing. Of the two “isms”, materialism — or physicalism—thus seems the most plausible starting point for the study of mental phenomena, as far as they are rooted in the brain, but without the negative connotations of “materialism” or “reductionism” in some modern philosophy of mind and popular writings.

The impact of Cartesian dualism has indeed faded over the centuries. However, covert dualism still appears to slumber in the popular view that the mind and brain, although distinct, can interact (Churchland, 1986, 2002; Valtonen et al., 2021), without specifying the physical implementation of the mind within the brain. For example, a popular metaphor is that the mind is software programmed “to make the brain work” (Dennett, 1991). These forms of “cognitivism” (Newell, 1980) relied on the computer metaphor, allowing researchers to study cognition and its computations without considering the underlying material brain and its neural systems (Kelty-Stephen et al., 2022). More importantly, perhaps, is the persistent argument raised by Nagel (1979) and Chalmers (1996, 2005) that we do not understand how an objective physical process could be sufficient for, or constitutive of, the subjective character of a conscious mental process. To state the problem this way, as the mental vs. the physical, situates it within the Cartesian framework and promotes the explanatory gap, without suggesting ways to resolve it. Finally, dualism has even shown up in neuroscience in the covert dualistic attitude manifested as the unwillingness to discuss neural mechanisms of consciousness, “leaving the problem of consciousness to psychologists and philosophers” (Arshavsky, 2006).

## 3 A theory of memory representations

The concept of representation has been employed in philosophy and in recent neuroscience literature, with definitions in the latter domain more closely aligning with the propositions presented in this article. This is particularly true because representations are essentially the brain's models of the external world and the body's states, allowing organisms to perceive, recognize, and interact with their environment. A clear definition in the recent neuroscience literature is that representation is “a key brain function in realizing a plethora of sensory, motor, and cognitive processes, some carried out consciously and others not” (Storm et al., 2024, echoing other recent definitions of consciousness from a neuroscience perspective).

A popular notion from the literature is that neural assemblies underlying memory representations contain copies or models of both the external and internal worlds (see Goldman-Rakic, 1987; Harnad, 1990; Deacon, 1997; Pereira, 1999; Spunt et al., 2015; Morgan and Piccinini, 2018; Giotakos, 2023; Heinen et al., 2024, for similar proposals). A related view is that representations can “project” these coded contents into our body or external world. An alternative suggestion in Pennartz's theory of neurorepresentationalism (NREP) is that the representational activity *itself* creates the conscious experience of intentionality as a virtual reality (Pennartz, 2015, 2022).^1^ Memory representations “built-in” internal modeling capacity interprets physical reality into subjective experience, similar to the interpretation of ambiguous information in an illusory visual object within the perceptual modality. In the following two sections, we will first present a definition and classification of memory representations and then describe how two significant types of memory representations can be distinguished in the subcortical-cortical pathways of the brain.

### 3.1 Definition and taxonomy of memory representations

We propose a definition of representations derived from a unified theory of long-term memory, encompassing both its explicit and implicit divisions (Squire and Zola Morgan, 1991; Squire, 1992; Sridhar et al., 2023; see Box 2). Evidence for multiple memory systems comes from behavioral dissociations. Lesion studies have demonstrated that certain types of brain damage impair only implicit performance, while others impair only explicit performance (Gabrieli et al., 1995).

Box 2 **Definition and properties of memory representations in the present article.**

• Memory-representations: knowledge elements of our memory system that vary in type (explicit, implicit), state (long-term, short-term), process (encoding, retrieval), and content (episodic, semantic, perceptual, motor).
• Explicit memory: knowledge elements to which we have conscious access, depending on medial-temporal structures in the brain.
• Implicit memory: knowledge elements to which we do not have conscious access, not depending on medial-temporal structures in the brain.
• Access consciousness: inherent capacity of encoded memory representations in long-term memory to generate conscious experience of information.
• Phenomenal consciousness: conscious experience of the content of representations during the retrieval of information from long-term memory, corresponding with the ‘what is like' aspect of consciousness.
• Affective content: Affective quality of memory representations through interactions between structures of explicit memory and the limbic system.
• Intentionality: inherent property of the content of memory representations to be about things or aspects of the environment.
• Coarse coding: refers to the broadly tuned character of representations residing in networks of the secondary association areas of the brain.
• Hierarchical modularity: a theory referring to large-scale networks where neural elements or modules are not only connected with other modules at the same coding level but also with elements of modules in successively higher-order levels, exhibiting increasingly integrative properties.
• Predictive coding: theory that the brain is a predictive organ, engaging in probabilistic computations of future events that require continuous updates based on actual evidence.

In addition to its integrative qualities, multiple memory theory offers a favorable perspective for reconciling different views in neuroscience and philosophy of mind, particularly concerning concepts such as consciousness, intentionality, and the “hard problem of consciousness”.

#### 3.1.1 Explicit vs. implicit memories

The theory of multiple memories, as introduced by Larry Squire and colleagues, describes the structure of long-term memory, distinguishing between declarative and non-declarative types. Declarative memories refer to those to which we have conscious access, whereas non-declarative memories do not. Non-declarative memory is sometimes referred to as procedural memory, or the memory of “how to do” things. Declarative memory is traditionally divided into episodic and semantic memories, with episodic memory referring to autobiographical events and events that are time and place-bound (Tulving, 1979; Schacter and Tulving, 1994; Sugar and Moser, 2019).

Semantic memory refers to general knowledge of facts and the meaning of concepts and language. It is often derived from episodic memory, gradually becoming “semantised” and losing its sensitivity for subjective aspects of time and place (see also Kiefer and Pulvermüller, 2012). A revision of the episodic-semantic division has further led to the insight that these two forms of memory are interdependent, and even included the “subjective experience as the central aspect of remembering that is to be explained and understood” (Greenberg and Verfaellie, 2010; Renoult et al., 2019; Rugg and Renoult, 2025).

Schacter (1995) later introduced a distinction between explicit and implicit memories, which became synonymous with declarative and non-declarative memories. These distinctions refer to memory contents we have conscious access to vs. those we do not. Here, we will mainly utilize the explicit-implicit distinction as umbrella terms, referring to their subcategories as forms of memory with specific content. For example, episodic memory is a subform of explicit memory characterized by its specific time and place-bound content.

Explicit and implicit memories are expressed differently, but they also posit fundamental differences in the neural assemblies that underlie their manifestations. Explicit memory representations have been assigned the property of representational flexibility, the “ability to be manipulated and used flexibly to guide performance in a variety of conditions” (Eichenbaum and Cohen, 2001, p. 168). In Schacter's terminology, implicit memory refers to various forms of memory, such as priming, conditioning, habit learning, and sensorimotor skills, that are assumed not to be directly associated with conscious experience. What they have in common is that they do not depend on consolidation by the medial temporal structure of the brain. For example, even a voluntary act like pressing a button could be carried out automatically in the motor system of our brain; conscious awareness occurs after the movement is executed. Implying that the subjective decision or “will” to move could be reconstructed according to a process of inference based on elements that come after the action (Banks and Isham, 2009; Dominik et al., 2017). ^2^

#### 3.1.2 Active vs. passive representations

It is generally assumed that different parts of the brain perform distinct functions, which may relate to the content of memory representations or context, such as the *where, what, when*, or *how* characteristics of memories. Here, we focus on another important aspect of memory: the activational state of the underlying neuronal assemblies, which is a crucial condition for conscious access to the content of memory representations.

**Figure 2:**
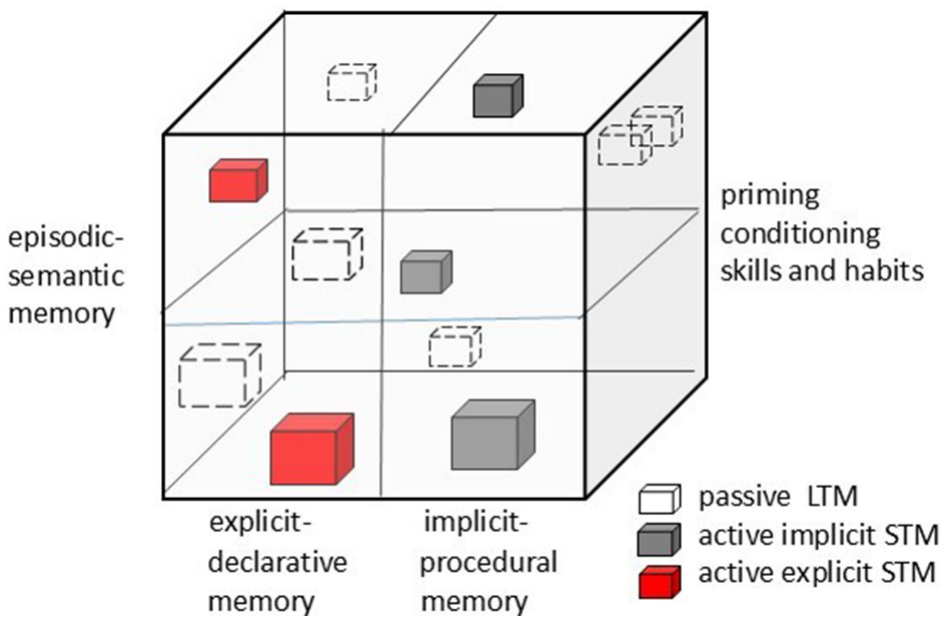
Schematic 3-D model of explicit versus implicit representations (left and right columns) with their respective subcategories listed on the left and right sides of the model. Specific memory representations reside in long-term memory (LTM, indicated by white stippled boxes) or are activated in short-term memory (STM, indicated by black and red boxes). Only boxes in the left column designate representations we have conscious access to (white stippled boxes) or that emerge as conscious experience (red boxes). Notice that in real brain space, representations correspond with networks, not with local structures, as could be suggested in our schematic model. See the following paragraphs for further details.

The schematic model in Figure 2 assumes that long-term memory (LTM) and short-term memory (STM) do not refer to qualitatively different structures or regions. Instead, they reflect different states (passive vs. active) of identical neural assemblies in cortical regions that function as repositories of information (Fuster, 1995; Kok, 2020). Passive representations refer to the dormant capacity of a network residing in the connection weights, implying that they are not directly accessible for conscious exploration (Churchland, 1986; Churchland and Sejnowski, 1992; Smith-Churchland, 2002). In contrast, active representations are brain activity patterns that “happen now” and represent the activated state of the same elements in STM networks. The distinction between passive and active reflects different positions along a brain-state continuum rather than a distinct dichotomy. For example, during learning, representations in the brain's network gradually transition from dormant to moderately activated to strongly activated, a process often considered necessary for conscious access.^3^

To align with the common terminology in learning and memory research, we will henceforth use the terms “encoding” and “retrieval” to designate the two successive stages of a learning process. Encoding refers to the initial learning phase, during which information is acquired and consolidated in memory. In the model depicted in Figure 2, they comprise the representations (depicted as white boxes) that, after encoding and consolidation, collectively constitute our LTM. Retrieval, in turn, refers to “utilizing stored information to create a conscious experience or to execute learned behavior, like a motor act” (Kok, 2020). In the present conceptualization, retrieval comes close to “reinstatement”, that is, the activation of the same area that is activated during encoding, although retrieval could also involve additional constructive processes (Sederberg et al., 2003; Johnson and Rugg, 2007; Rugg and Renoult, 2025; Eichenbaum, 2012; Lee et al., 2013; Preston and Eichenbaum, 2013). ^4^ Retrieval should be further understood as any factor capable of reinstating or “igniting” a dormant network in STM (Aronowitz, 2018). This could include an external salient event, performing a cognitive or motor task, voluntary attention, spontaneous thoughts, and mind wandering. Current models of conscious access have indeed proposed that sufficiently strong stimuli—and concurrent strong neural activations—are needed to ignite a global network of regions, allowing further processing and the experienced content of information (Tagliazucchi, 2017). The same principle was formulated even more concisely by Pennartz as: “sensory signals, at first being “preconscious”, must pass a threshold of ignition that unleashes the broadcasting of activity” (Pennartz, 2022).

Another important aspect of the model presented in Figure 2 is that conscious awareness (or conscious experience) of events occurs only for retrieved representations in explicit STM. In contrast, retrieved representations in implicit STM memory space do not allow conscious access.^5^

#### 3.1.3 Access consciousness and phenomenal consciousness

In the model sketched in Figure 2, conscious experience depends on two conditions of memory representations in explicit memory. First, information must be fully encoded or consolidated in LTM; second, the retrieval of the content of encoded memory representations in STM will generate conscious experience. The necessity of these two conjunct conditions for conscious experience is reminiscent of a hotly debated issue in philosophy of mind. This concerns the validity of the distinction between “access consciousness” (AC) and “phenomenal consciousness” (PC). The argument is that the mental content accessed is not (always) identical to the content experienced (Block, 2007; Overgaard, 2018; Fahrenfort and Lamme, 2012). Following the dual mechanism view, we propose that AC refers to the encoded and stored contents of representations in explicit long-term memory that possess the inherent capacity or potential to generate conscious experience (as depicted by the white boxes on the left in Figure 2). In turn, PC refers to retrieving encoded content to create the experienced representation (depicted as red boxes on the left in Figure 2).^6^ In contrast, encoded and stored content of representations in implicit memory (white boxes at the right in Figure 2) does not allow AC, and by definition, also no PC. Retrieval of stored content from implicit long-term memory representations (the gray boxes on the right side of Figure 2) will be expressed differently—for example, in executing learned skills and motor activities—depending on brain circuits that do not require or involve conscious access. A factor worth considering, however, is that although profound neurocognitive differences exist between explicit and implicit memory, implicit memory can influence behaviors typically associated with explicit memory (see Voss et al., 2012, for examples).

### 3.2 A functional network model of memory representations

Memory representations, as introduced schematically by their content and state, refer to the “knowledge elements” of both the outer and the inner world, as well as to anticipations and the execution of overt actions (Kok, 2020). They are linked to distributed networks within the brain, connecting local modules via long-range reciprocal pathways. These networks appear primarily in the anterior and posterior cortices, connecting to subcortical areas. Together, they constitute a significant portion of our functional cerebral space, encompassing various information-processing functions that utilize memory representations. The content of memories can be expressed as conscious awareness or not, depending on the involved type of memory and the corresponding activating neural ensembles.

#### 3.2.1 Thalamic nuclei and pathways

**Figure 3:**
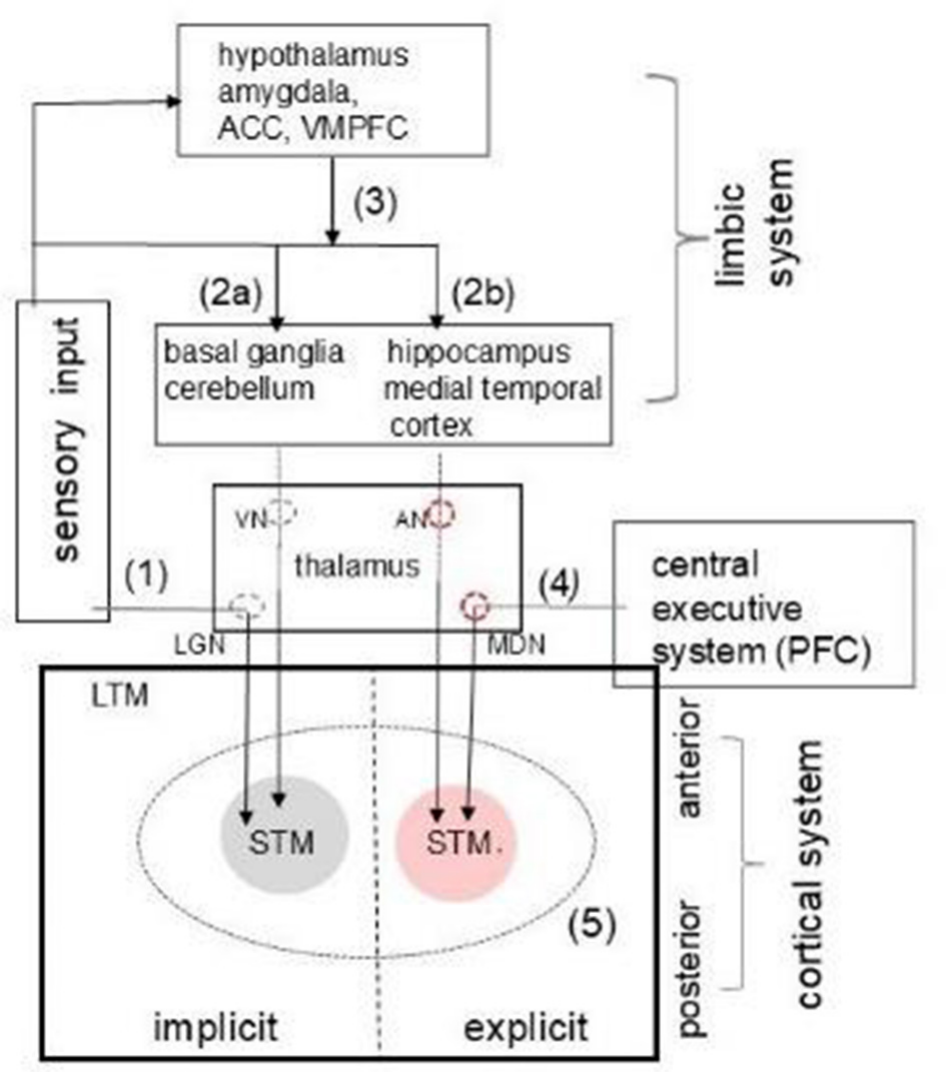
Sketch of our memory model with various routes to activate memory representations. Thalamus: VN, ventral nucleus; AN, anterior nuclei; LGN, lateral geniculate nucleus; MDN, medial dorsal nucleus. Activation of short-term memory (STM) is presumed to occur in a subset of passive long-term memory (LTM) via three different thalamocortical routes. (1) Directly from sensory input and thalamus (priming), (2a) input from structures in the cerebellum and basal ganglia, (2b) input from hippocampus and medial temporal cortex, (3) modulatory input from affective limbic structures to the basal ganglia and medial temporal cortex, (4) top-down input from the central executive system in the prefrontal cortex. (5) overarching network integrating active implicit and explicit memory representations in STM. See text for further clarification.

Figure 3 illustrates in detail the various structures and routes of the long-range pathways in our memory model, with the thalamus serving as a central node to the cortex. Contrary to common belief, most of the input to the thalamus does not come from sensory organs, but rather from the brain itself. Structural connections between networks in the brain need to be “powered” or “ignited” to become functional. In the configuration depicted in the model, the thalamus occupies a strategic position to perform such an “enhancing” role in the involved pathways (denoted respectively as 1, 2a, 2b, and 4). Some nuclei in the thalamus (like the lateral and medial geniculate nuclei) function as stations relaying sensory information via long recurrent fibres to the primary areas in the cortex. Notably, the thalamus also contains nonspecific nuclei, such as the pulvinar and medial dorsal nuclei, which connect to the association areas in the cortex (LaBerge, 1995; Whyte et al., 2024). In this respect, the thalamus is the principal gateway to consciousness, modulating or enhancing the state and content of memory ensembles (LaBerge, 1995; Whyte et al., 2024). The anterior thalamic nuclei (AN) are significant to our memory model and are consistently associated with the consolidation of explicit memory representations. They form a triangular network with the hippocampus and the temporal cortex (Aggleton and O'Mara, 2022).

In contrast to the AN, which is part of the anterior dorsal thalamus, the ventral-anterior nucleus (VN), located in the lateral thalamus, connects the basal ganglia with the premotor cortex. The specific configurations of these two subcortical systems, which project via different thalamic nuclei to distinct parts of the cortex, may also provide insight into why some mental representations in the brain give rise to conscious experiences, while others do not.

#### 3.2.2 Thalamic cortical loops^7^

The following routes are described in Figure 3.

Route 1 refers to priming, a form of implicit memory defined as the ability to respond to a stimulus following prior exposure. This route projects to the primary sensory areas in the cortex via the corresponding relay nuclei in the thalamus.

Routes 2a and 2b refer to thalamocortical routes originating in different subcortical regions of the brain and controlling implicit and explicit forms of behavior. They represent recurrent or re-entrant pathways, as defined by Gerald Edelman (Edelman, 1992; Edelman et al., 2011; Edelman and Gally, 2013). Connection via recurrent pathways is in itself not a sufficient condition for generating conscious experiences. This requires additional selective recruitment of neural assemblies, mediated by reciprocal long-range pathways from the medial temporal hippocampus (Figure 3, pathway 2b).

The heterogeneity of the basal ganglia is also reflected in differences among the pathways that project to the cortex (collectively designated as route 2a in Figure 3). Although these pathways in general will not generate conscious experiences, some connections between the dorsal striatum projecting to premotor areas in the cortex also seem to reflect the mechanism of long-term potentiation (LTP) of the hippocampus (Doyon and Ungerleider, 2002; Eichenbaum et al., 2012). Other, more elementary types of implicit memory controlled by the striatum and thalamus, such as habit learning, are probably associated with simple associative forms of synaptic transmission (Knowlton et al., 1996). Finally, the ventral striatum of the basal ganglia, also known as the nucleus accumbens, is primarily involved in regulating affective forms of behavior and reinforcement learning, utilizing the dopamine pathways (Kok, 2020; Sesack and Grace, 2010; Rusu and Pennartz, 2020). Here, the involved pathways play a specific role in the interaction between subcortical structures (basal ganglia and medial temporal cortex) and the limbic system, mediated by route 3.

Route 3, in the upper part of Figure 3, recruits various limbic system structures, including the amygdala, the ventromedial prefrontal cortex (VMPFC), and the anterior cingulate cortex (ACC). Interaction between these structures and the memory systems can occur in two different ways. In the explicit version, limbic structures, such as the amygdala, modulate explicit memory formation via the medial temporal cortex. In the implicit version, they are the core structures for establishing memories that are part of reinforcement and skill learning, independent of explicit memory (Eichenbaum and Cohen, 2001; Emery and Amaral, 2002; see for further details section 5).

Route 4 is mediated “top-down” by prefrontal cortex regions, functioning as the “central executive”. This modality non-specific system of limited capacity is functionally equivalent to working memory (Baddeley, 2003). In Figure 3, the primary function of the central executive system is to manipulate and retrieve information stored in short-term memory. This involves a pathway running via the medial-dorsal nucleus (MDN) of the thalamus to the prefrontal cortex, in parallel with (or interacting with) a pathway that runs from the medial temporal cortex via the anterior nuclei (AN) of the thalamus to the temporal association cortex. Since working memory involves consciously accessible information, it is generally assumed to be closely related to consciousness (but see Hassin et al., 2009 for exceptions). A relevant finding in this context is that extensive practice of tasks in the domains of skill learning and declarative learning does not alter the general architecture of the structures and pathways of explicit and implicit memories. Instead, it could lead to reduced demand on the mechanisms controlling neural assembly states in the later phases of learning. In the configuration of pathways illustrated in Figure 3, this implies a gradual decline in top-down input from the PFC to the STM during prolonged practice.^8^ Additionally, the available literature indicates that the encoding and retrieval of content in explicit memory involve a dynamic interplay between the prefrontal cortex and specific hippocampal regions (Nyberg et al., 1996; Hassin et al., 2009; Wiltgen et al., 2010). The process of updating contextual knowledge based on new learning, in particular, could be mediated by the ventral medial prefrontal cortex (Preston and Eichenbaum, 2013). In the framework of the model depicted in Figure 3, these dynamic scenarios would involve a network comprising routes 2b, 4, and 3.

*Section 5*, located in the lower part of Figure 3, refers to an overarching network that integrates implicit and explicit memory representations. As suggested earlier, the separation between explicit and implicit memories does not imply that these memory systems are functionally independent. Tasks performed in daily life often depend on complex skills that integrate aspects of perceptual, motor, and explicit memory functions (Turk-Browne et al., 2006). A concrete example is familiarity memory, which seems particularly prone to the accidental capture of implicit memory processing (Voss et al., 2012). The interactive principle was also emphasized by Reder et al. (2000), arguing that “some implicit and explicit memory tasks share the same memory representations, and that the important distinction is whether the task (implicit or explicit) requires the formation of a new association”.

### 3.3 Conclusions

This chapter provides a detailed description of the various structures and pathways underlying both explicit and implicit memory. It also provides arguments for how these two distinct forms of memory can interact. We here present a summary of the factors justifying their distinction and interaction.

Conscious access or not. Several factors may explain why explicit and implicit memories give rise to conscious access or do not. Why is implicit memory triggered without conscious awareness? Perhaps the most obvious explanation is that various forms of implicit memory share the commonality that they do not depend on the medial temporal cortex. This criterion has also proven to be a powerful tool for distinguishing performance domains where amnesic patients succeed and fail. A more substantive argument is that behaviors dependent on goal-directed learning of action-outcome contingencies and reinforcement-based learning would inherently depend on the biologically efficient and fast striatal-cortical pathways. Memory representations controlled by these pathways would likely remain “dispositional” (Damasio, 1994, 1999), that is, not directly dependent on conscious experience or internal models of the environment or action repertoires. In addition, intended actions realized by these pathways usually have the character of routine skills triggered without the intervention of the retrieval mechanism of the prefrontal cortex (Goschke and Kuhl, 1996). Research has further shown that in dual-task performance, motor skills do not interfere with tasks requiring conscious control (Ewolds et al., 2017), supporting the view that they do not rely on the limited capacity of consciously controlled brain structures. The same argument holds for the structures and routes of reinforcement-based learning, which regulate affective and “instinctive” forms of behavior via the dopamine pathways. In short, they utilize biological routes shaped by evolution to serve the immediate goals of survival.

Integrating implicit and explicit memories. Implicit-explicit memory interactions are often integrated as they occur in our daily performances. Unlike controlled processes, implicit and automatic functions do not require limited processing resources. This entails that they can be appended to functions without interfering with their control in systems with limited capacity, such as working memory. For example, driving a car in normal traffic conditions (typically a sensorimotor skill) can be time-shared with a conversation with your passenger (typically depending on explicit memory). Another spin-off of the fusion of implicit and explicit functions is that implicit memories, such as skill learning and priming, become available for conscious exploration (Dayan and Cohen, 2011). This will be especially true in tasks that involve elements such as providing feedback on performance, detecting errors, and identifying mismatches between novel stimuli and representations in long-term memory.

### 3.4 Related theories

The Theory of Global Workspace (TGW) has attracted significant interest among researchers in neuroscience and cognitive psychology. In short, this theory states that top-down attention is a prerequisite for global workspace neurons to become conscious (Baars, 1988, 2003; Dehaene et al., 1998; Dehaene and Naccache, 2001). The global workspace links distant areas of the brain, including the prefrontal cortex, which inhibits surrounding neurons outside the workspace. Our model shares some similarities with TGW. For example, implicit sensorimotor skills may gain conscious access when they are fused with explicit memory within a multilevel representational network. On the other hand, in our model, conscious access and conscious experience occur within the broader context of representations retrieved from explicit long-term memory via routes 2b and 4. Importantly, our model also incorporates a principle lacking in TGW: intentionality, the capacity of elements in memory representation to interact with the external world (see section 6.2 for a further discussion).

The multimodal multilevel network model proposed by Pennartz (2022) suggests that conscious representations emerge from low-level neuronal configurations at the top of a hierarchical network. His model, however, does not seem to differentiate between explicit and implicit forms of learning and memory and would (in our view) apply only to the multilevel network of the hippocampal-cortical loop, creating full, conscious representations of objects integrated in space. Our model, on the other hand, allows for the differentiation of explicit and implicit forms of learning, both of which utilize the architecture of multimodal hierarchical structures. For example, an activated memory representation of a complex skill, such as catching a ball from the air by a professional baseball player, would not necessarily be accompanied by conscious experience, even if it were at the top of a hierarchy recruiting low-level neural configurations in the basal ganglia. The only route for implicit representations to reach conscious access is through their “fusions” with elements of explicit memory in an overarching representational network as sketched in Figure 3.

In the following paragraphs, we will elaborate on which neural principles underlie the computational capacity of memory presentations to interpret or “model” aspects of the outside world (as briefly introduced in section 3). We first sketch how representations may emerge in multilevel hierarchical networks—from local networks with precise coding to more global networks with increasingly coarser coding forms—and how these representations give rise to conscious experience in the association cortices. Prediction coding, then, is assumed to provide the representations at the top of the hierarchy with the mechanism for interpreting or modeling properties of the outside world. Our second focus is on another keystone of explicit memory, namely, how the content of episodic memories acquires a subjective-affective quality through interaction with structures in the limbic system.

## 4 Coding in neural assemblies underlying representations

A great deal of knowledge has been gathered about the neural circuits that mediate various forms of memory. However, a central but still unresolved problem is understanding *how* the content of representations is stored and utilized in the neural assemblies and neurons of the brain. This involves gaining insight into the computational properties of representations, particularly those that fall under the umbrella of the explicit memory system. We discuss several aspects of the coding problem in successive order.

Ensemble coding, Ensemble or (or “vector”) coding seems to be a plausible mechanism for storing and retrieving memory representations, particularly in distributed networks of the temporal cortex, where cell selectivity is always relative, not absolute. The nature of these coding principles remains a matter of speculation. However, it ultimately comes down to each cell knowing which partners are most attractive to connect with, using relational codes.

**Figure 4:**
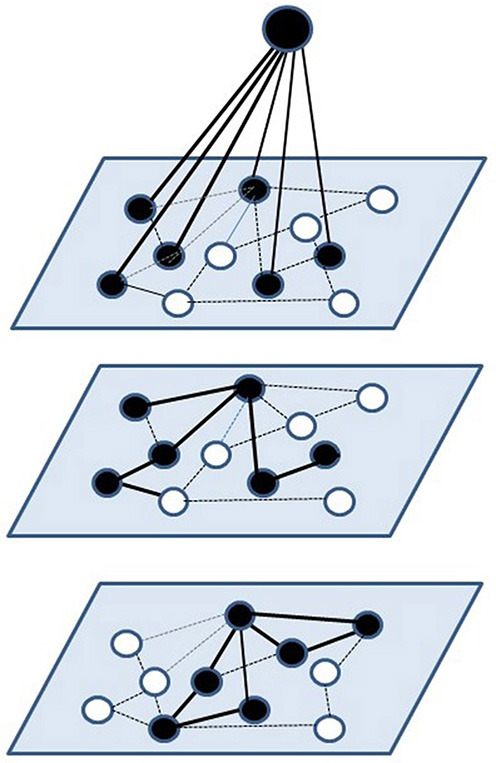
Schematic illustration of two coding principles in a neural assembly. Upper panel: a separate binding unit links elements in a network. Middle and lower panels: ensemble coding with neural elements forming distinct yet partially overlapping alliances within the same structural network; see the text for details. Adapted from Kok (2020).

Ensemble coding (Figure 4) is a principle that applies to memory representation in the sensory modalities, as well as to action and motor representations (Rizzolatti and Craighero, 2004; Whitney and Leib, 2018; Aery Jones and Giocomo, 2023). Specifically, action representations have been described as distributed networks spanning the posterior and anterior regions of the cortex. In this configuration, a motor sequence could take the form of motor programs, which code for the successive spatial coordinates of motor trajectories, involving long-range connections between the parietal and premotor cortices (Fuster, 1995; Rizzolatti et al., 1996; Tranel, 2003).

Representations can also have a dual or conjunctive content, meaning that the context in which events occur can be bound into a unitary representation, with the hippocampal formation assigned an important role in encoding and storage of conjunctive representations (O'Reilly and Rudy, 2001). Conjunction can refer to various features of an object, different modalities, and even affective content added to explicit memories (see section 5).

A challenging question is how the identical neural assemblies that store information can also interpret the content of memory representations into subjective phenomenal experiences. A tentative scenario is that neurons in distributed networks in the brain will gradually increase their receptive fields during learning, leading to the formation of more coarsely coded, yet richer, multimodal information. Concurrently, the hippocampus, targeting neurons in these assemblies via reciprocal pathways, will begin binding “where” and “what” information streaming in the dorsal and ventral regions of the posterior cortex, thereby increasing the size and complexity of the cortical assemblies. The resulting encoded episodic memory representations residing in the inferotemporal cortex will then become available for retrieval, which, in turn, will trigger or “ignite” phenomenal experiences. In our model of memory representations, these two stages are presumed to utilize the hippocampal system, in concert with parts of the prefrontal areas, depending on the specific form of retrieval (Tulving, 1994; Spaniol et al., 2009).

Hierarchical modularity. Influential theorists have assumed that large-scale networks, as just sketched, follow the principle of hierarchical modularity (Felleman and Van Essen, 1991). In such a neural configuration, neural elements or modules are connected to other modules at the same coding level and to elements of modules at successively higher-order levels, exhibiting increasingly integrative properties. ^9^ Pennartz (2022) proposed a similar theory of functional organization of different representation levels in constructing a visual object's conscious experience. This is achieved through a bottom-up process, in which successive levels of single neurons form small local networks that are combined at subsequent levels into unimodal and multimodal multilevel networks, thereby creating full, conscious representations of objects integrated in space.

The principle of coarse coding in large hierarchically organized networks is also compatible with the earlier notion of representations being not mere copies, but “best guess” interpretations of neural aggregates of broadly tuned neurons, which could sometimes lead to errors in recognition. For example, when we mistake the face of an unfamiliar person on the street for that of a friend. In the motor domain, coarse coding is also the strategy the brain uses to create representations. This is exemplified in how the motor cortex represents the direction of movement. For example, motor neurons in the monkey brain have been shown to exhibit broad tuning in many individual neurons, specifically in the direction of movement. However, the population vector's average output corresponded accurately with the actual direction of each movement (Georgopoulos, 1995; Cowper-Smith et al., 2010).

Predictive coding in multimodal hierarchical networks—the representational mechanism capable of interpreting or modeling properties of the outside world—is presumed to reside in explicit memory representations atop the hierarchical network. Such a mechanism has been formulated in terms of predicted coding or predictive processing (Millidge et al., 2021; Sprevak and Smith, 2023). These abilities of the brain, although seeming highly sophisticated, likely emerged gradually from simpler predictive loops, such as automatic motor actions or error-detection subroutines (Pezzulo et al., 2021). Predictive coding, however, refers not so much to predicting future events as to comparing the effects of internal representations with “here and now” sensory inputs, thereby allowing the computation of a prediction error, which subserves both perceptual learning and inference (Storm et al., 2024).

The predictive coding principle is closely related to template matching, neuronal models, and comparator mechanisms (Barlow, 1994), and it requires continuous updates based on evidence (Darriba and Waszak, 2018).

**Figure 5:**
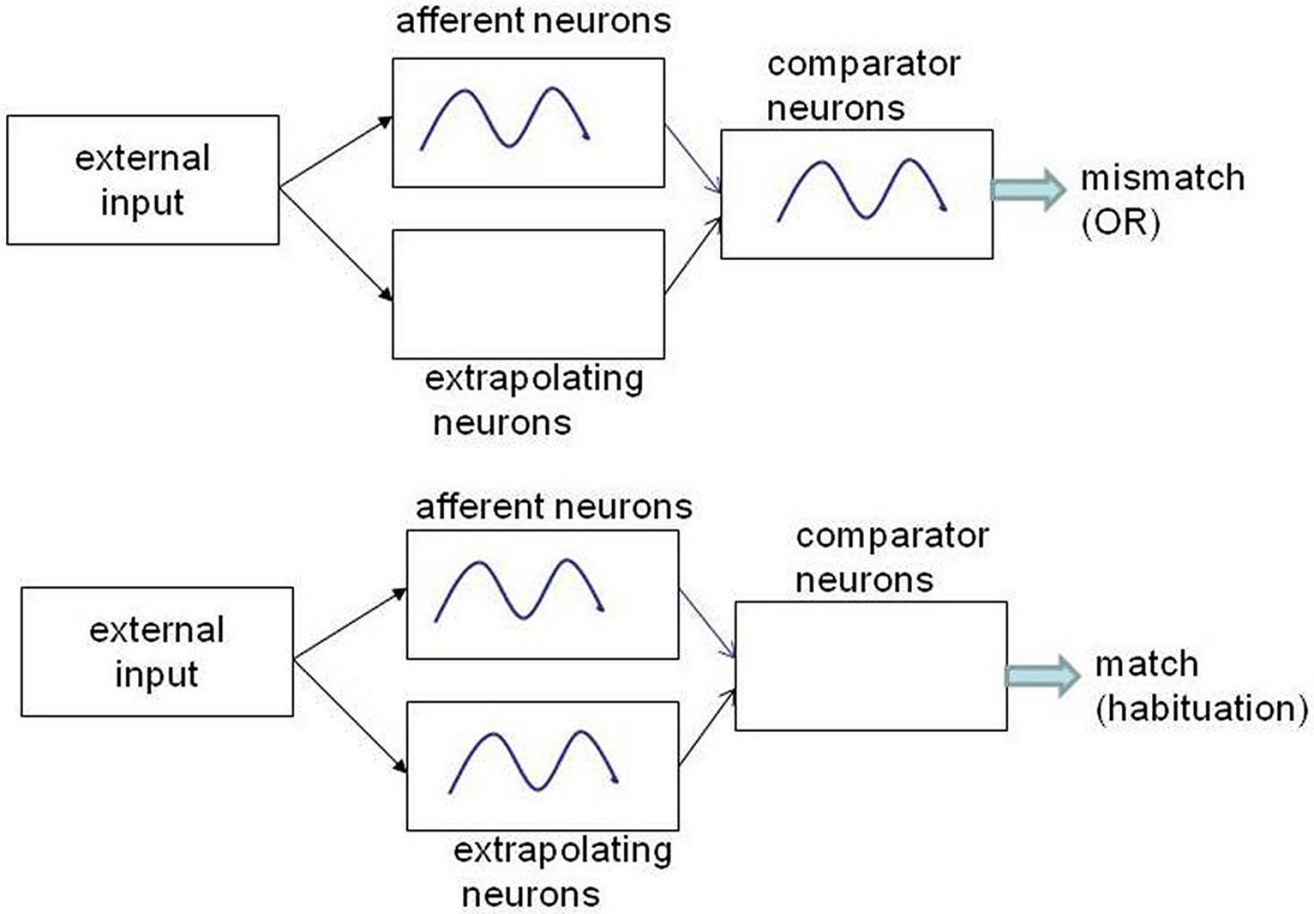
Model of the orienting response as initially proposed by Sokolov. External input is sent to afferent and extrapolation neurons, which in turn send output to comparator neurons, eliciting either a mismatch (upper panel) or a match (lower panel. See the text for details (adapted from Kok, 2020).

Several propositions have been made regarding neurocognitive systems that accommodate predictive coding and neural models, which we will discuss briefly here.^10^ Jamous and colleagues suggested that during the integration of perception and motor codes, theta-band activity in the insular cortex and temporo-hippocampal structures is modulated by the predictability of upcoming information (Jamous et al., 2024). Neuronal model and comparator mechanism are also incorporated in the orienting response model, formulated by Sokolov (Van Olst, 1971; Sokolov and Vinogradova, 1975). In this model, comparator neurons (functioning as a “mnemonic” filter) fire in response to a mismatch between afferent and extrapolation neurons. Neuronal models, in turn, would gradually build up during repeated presentations of the same stimulus, a phenomenon known as habituation (see Figure 5). Mismatches caused by highly significant or novel events can pass the mnemonic filter, allowing further access and exploration in consciousness. Later studies have suggested that neurons in the hippocampus could function as familiarity-novelty detectors, similar to comparator mechanisms. This would also align with the proposed roles of the hippocampus and the adjacent entorhinal cortex as temporary buffers for storing unstructured information, complementing the neocortex's long-term storage of explicit memories (Kumaran and Maguire, 2005, 2007). ^11^

## 5 Affective modulation of memory representations

Cognitive neuroscience has also developed a theory specifying how “cognition” (the knowing and reasoning part of the brain) and “emotion” (the feeling part of the brain) interact with one another (Lane and Nadel, 2002; Hudlicka, 2017; Kok, 2022; Perrotta, 2025). This included the proposal that memory theories should focus more strongly on the subjective elements of memories (Greenberg and Verfaellie, 2010; Renoult et al., 2019; Rugg and Renoult, 2025). In addition, philosophy of mind has recently adopted the idea that the phenomenal quality, also referred to as the “feeling part, ‘qualia, or 'what it is like” aspect of consciousness, supervenes on representational content (Shim, 2011; Tye, 2000).

A key aspect of our theory of memory representations, briefly discussed in the introduction of our model in Figure 3, is the role of emotion in regulating human memory. Experiences involving emotionally significant events are far more likely to be successfully consolidated into a long-lasting memory than are less emotionally significant events. Significantly, emotions create a highly subjective “qualitative” content of episodic elements of explicit memory. Structures in the limbic system, including the amygdala, anterior cingulate cortex, and ventromedial and dorsolateral prefrontal regions, function as an integrated affective network (Bush et al., 2000). In this integrative network, the anterior thalamic nuclei have been suggested to play a role in emotional control in concert with the orbitofrontal cortex and the anterior cingulate cortex (Sun et al., 2015, see also Figure 3).

Affective modulations of memory representations can occur in two different ways. In explicit memory, affective modulation occurs through interactions between the basolateral amygdala (BLA) and hippocampal-dependent declarative memory. In this circuitry, BLA mediates the emotional significance of experiences by modulating the consolidation processes, concurrently enhancing the strength of the resulting memory representation (Allen et al., 2008; Richter-Levin, 2004; Kok, 2022). Adrenaline released by the amygdala, affecting synaptic connections with the hippocampus, could further contribute to the enhancing effect on memory representations (Emery and Amaral, 2002). In contextual fear conditioning, a process assumed to be also mediated by the hippocampus-amygdala interactions, rodents display a natural defensive response termed freezing, for example, to the situation or place in which the administered shock took place (O'Reilly and Rudy, 2001).

In implicit memory, the encoding of affective events occurs without conscious awareness, utilizing principles of Hebbian learning, which does not require the intervention of the hippocampus or the medial temporal cortex (LeDoux, 1996). Examples are circuits of reinforcement guided learning, coupling the ventral striatum, anterior cingulate cortex, and orbitofrontal cortex with the reward value of stimuli in the macaque (Rushworth et al., 2011), or with rewarding stimuli in a discrimination reversal task in normal subjects and patients (Rolls, 1999, 2000, 2008).

In sum, the dense interconnectivity between the brain's affective and cognitive (i.e., memory-related) networks implies that the affective content of stimuli can “penetrate” and “color” memory representations. This is achieved through long-range connections between the limbic system and posterior cortical regions. The resulting memory-affective interplay underlies the subjective phenomenal content of representations during the encoding and retrieval of information in episodic memory and reward-driven forms of implicit learning.

## 6 Solving the “hard problem”

The present section of the article will delve deeper into how subjectivity and personal experiences are rooted in the brain, focusing on terminology and concepts from the philosophy of mind. Together, they focus on how subjective personal experiences are related to the brain's physical structures. Chalmers (1995, 1996) formulated the same question as the “hard problem in consciousness”, alias the problem of experience accounting for “what it is like” or “qualia” aspects.

Tucker et al. (2022) stated that insight into the “hard” problem can be gained by integrating classical phenomenological studies of experience with recent progress in differential neurophysiology, specifically the consolidation of explicit vs. implicit memory. In addition, Dennett (2018) proposed approaching this problem from a different pragmatic angle, stating that Chalmers was “mis-focusing our attention, exaggerating an artifact of inquiry … the failure to ask, and answer, what I called the hard question”.

Following these suggestions, our starting point in this article was the definition of representations in terms of the unified theory of long-term memory, which can be interpreted as a cognitively inspired form of representationalism. We argued that, in addition to its integrative qualities, the theory offers a favorable perspective for bridging gaps between different views in cognitive neuroscience and the philosophy of mind. This holds particularly for concepts such as consciousness, intentionality, emergence, and qualia, which we have already briefly introduced in previous sections of this article.

Several areas, which, according to the available literature, reflect controversies between philosophy and neuroscience (see Figure 1), proved less contentious in our model of memory representations. In fact, as our model gradually unfolded, concepts such as intentionality, emergence, and even “what it is like” (qualia) definitions proved to fit rather well within our theory of memory representations. A similar trend was evident in recent influential neuroscientific reviews, which focused on the theoretical aspects of consciousness and neural representations (Storm et al., 2024).

Therefore, the strategy we follow in this chapter is not to confront the controversial issues between philosophy and neuroscience, but rather to explore the philosophical roots of concepts related to consciousness that could be incorporated into our model of memory representations. We start with consciousness, the most debated and controversial issue in philosophy of mind and neuroscience.

### 6.1 Consciousness

*Once we appreciate all the non-mysterious ways in which the brain can create benign “user illusions”*,^12^ *we can begin to imagine how the brain creates consciousness (Dennett, 2005)*.

In its simplest definition, consciousness is not problematic at all. It means being aware, or “knowing,” of objects or states, either internal to oneself or in one's external environment (James, 1981). Consciousness can manifest itself in three distinct ways: as a state of alertness or wakefulness, as a process of orienting to significant events, or as an internally controlled process, such as thinking or deliberately focusing on a specific attribute when stimulated or faced with a demanding task. These various manifestations are a product of a hierarchical thalamic-cortical system with different levels controlling different manifestations (Kok, 2020; Whyte et al., 2024).

Philosophy of mind relies strongly on the introspection of phenomenal experiences, which is something within one's mind that becomes “introspectively conscious” (Chalmers, 1995, 1996, 2005; Dehaene, 2019). These insights and definitions align with aspects of the “explanatory gap” (Levine, 1983, 2009), a concept discussed earlier in this review. In the philosophy of mind, the phenomenal contents or the so-called qualia of consciousness are inherently subjective attributes of experience. By most accounts, they are considered non-representational in the physical sense (see Korf, 2014, for a review).

Challenging these views, Dennett (2011) argued that any neurobiological theory based on an experience/function division cannot be empirically confirmed or falsified and is thus outside the scope of “science”. Can we then develop falsifiable theories of consciousness grounded in the brain's properties? The answer is: yes. One example is neurocognitive theories of selective attention, as elaborated by LaBerge (1995). In addition, various studies with a predominant neuroscience orientation have recently presented an integrative, multiscale view on neural theories of consciousness (Flanagan, 1992; Lamme, 2010; Ellia and Chis-Ciure, 2022; Seth and Baine, 2022; Storm et al., 2024; Whyte et al., 2024; Mudrik et al., 2025, for reviews).

A theory that has attracted considerable interest in philosophy of mind and neuroscience is based on the global workspace, introduced earlier (Baars, 1988; Dehaene and Naccache, 2001; Dehaene et al., 1998). This theory describes the processing requirements that give rise to conscious experience as a dynamic interaction between local processors and a global workspace. Lamme and colleagues offer a different theoretical perspective on consciousness, based on research into primate vision (Lamme, 2010; Lamme and Roelfsema, 2000). They stated that recurrent or reentrant processing (RP) is essential and perhaps sufficient for visual conscious experience. In their extensive review of consciousness, Storm and associates commented on RP by suggesting that RP alone is insufficient for consciousness (Storm et al., 2024). An alternative recent theory, already briefly discussed in our model of memory representations, is neuroresentationalism (NREP), formulated by Pennartz (2015, 2022). NREP proposes that consciousness, in the sense of conscious experiences, may emerge on top of a hierarchy of conceptually distinct levels, with single neurons at its base and progressing upward through assembly-level, unimodal, and multimodal network representations. It was further proposed that representation is “a key brain function in realizing a plethora of sensory, motor, and cognitive processes, some carried out consciously and others not” (Storm et al., 2024, echoing other recent definitions of consciousness from a neuroscience perspective).

Our approach was not to introduce a comprehensive new theory of consciousness. Instead, we followed the tradition in the memory literature to re-frame consciousness as a “mode” of implicit vs. explicit processing, associated with a variety of information processing functions such as perception, memory, motor functions, and emotions (Kok, 2020; Cohen and Dennett, 2011; Tulving, 1985; Squire and Zola Morgan, 1991; Eichenbaum and Cohen, 2001; Sridhar et al., 2023). Within this framework, we defined conscious experiences as the product of two mechanisms: conscious access, which occurs through the process of encoding and consolidation of new information, and conscious experience, which results from the retrieval of encoded information in explicit long-term memory. In the same framework, retrieval incorporates any condition that would ignite slumbering representations in long-term memory, such as significant external events, task performance, but also spontaneous thoughts and mind wandering (Christoff et al., 2016).

In conclusion, the neurobiological processes that underlie conscious processes and subjective states are efficient but imperceptible, in contrast to the transparency of our subjective experiences. The “price we pay” for this biological efficiency is thus that we cannot understand the brain's inner workings through introspection (Musacchio, 2005). Still, the physical embodiment of information in the brain, reflected in patterns of action potentials, is intimately tied to information messages. The meaning of these messages could lie at the very basis of qualia.

### 6.2 Intentionality

In the tradition of dualism, philosophers of the mind have long struggled with the problem of intentionality, a concept introduced by Franz Brentano in the late nineteenth century (Textor, 2017). Intentionality is the power of minds and mental states to be about things, in short, that a subjective mental state includes something as an object within itself (Dennett, 1996; Mendelovici, 2018; Mendelovici and Bourget, 2023). Furthermore, stating that an individual's mental states have intentionality amounts to saying that they are mental representations or have contents. In philosophy, there is a tight connection between concepts such as intentionality and representation, which are discussed in more abstract terms within the overarching Representational Theory of Mind (Tye, 2009; Lycan, 1996).

The position taken in this article is that intentionality is a fundamental element of consciousness, residing in neural assemblies that incorporate representations. Representations harness the capacity of neural assemblies to predict or interpret events in the external world, utilizing the built-in modeling capacity of memory representations to transform physical reality into subjective experience (see also Pennartz, 2015, 2022; Giotakos, 2023). They underlie perceptual discrimination, memory, awareness, preparation of motor activity, and imaging of familiar visual scenes. Similar views were expressed by Alan Baddeley (2003), who referred to a “visuo-spatial sketchpad”, and by Anton Damasio, who spoke of “dispositional representations”, which are not raw copies of the outside world, but rather the potential of ensembles of neurons in the brain to fire and reconstruct or model the original events (Damasio and Damasio, 1994).

Intentionality also refers to how our thoughts and language are represented in the mind, with semantics concerned with the meaning encoded in language itself (Brandom, 2014). In this context, binding theories suggest that semantic knowledge involves a broadly distributed network of neural representations, particularly involving the inferotemporal and posterior inferior parietal regions. These regions enable increasingly abstract, multimodal representations of perceptual experience, supporting a variety of conceptual functions.

In summary, the theory of memory representations outlined earlier has the potential to incorporate the notion of intentionality, as elaborated in the philosophy of mind.

### 6.3 Emergence and qualia: the heritage of phenomenology

In philosophy, emergence often means irreducibility. The idea is that although mental properties arise from and supervene on the brain's physical properties, they can never be reduced to them. The concept formed a part of Roger Sperry's account of mind-brain interaction in his theory of emergent interactionism (Chezik, 1990). Neuroscientists have recently proposed that these processes emerge from hierarchical structures in complex systems, in which lower levels combine to form higher levels. New features emerge in the system as more levels are added or local assemblies in the brain become integrated into a much larger network with long-ranging connections (Feinberg and Mallatt, 2019; Dehaene, 2019).

Integration, in this sense, is a somewhat abstract principle that does not touch the richness and subjective character of our conscious experiences. A conscious experience can often be a vivid sensory image accompanied by a feeling, suggesting that your frontal cortex retrieves a slumbering subjective representation from visual memory. In other words, the subjective nature of consciousness depends entirely on the momentary locus of control within our representational systems (Donald, 1991). In episodic memory, consciousness is mostly situation-bound and concrete, with the locus of control at the top of the episodic system. Emergence in this context comes close to the content of phenomenal consciousness, in short, to “qualia”.

Qualia and memory representations. Philosophical discussions on the nature of intentionality have focused more strongly on the connection between intentionality and phenomenal consciousness, emphasizing that “intentionality is none other than phenomenal consciousness, the subjective, felt, or qualitative aspect of mental life” (see Kriegel, 2013a,b). In philosophy of mind, subjective conscious experiences, also defined as “qualia, are a core element of the hard problem of consciousness”, explaining how physical processes in the brain give rise to qualitative feelings like the redness of red. Chalmers (2005) argues that subjective experiences are fundamental to reality and may not be fully explained by purely materialist or reductionist models. Conscious perceptual experiences are, by essence, private and subjective; when I look at the blue sky with clouds, it can trigger a specific feeling that is difficult to describe and share with others (Searle, 2000). Similarly, Dennet (2002) defined qualia as “ineffable, intrinsic, private, directly apprehensible properties of experience” memory.

These definitions are heavily influenced by a traditional, strict version of phenomenology, initially formulated by Edmund Husserl, who posited that a perceived object or event should be described precisely as it is perceived. Husserl used the word “hyletic”, meaning “about matter, the raw, material data of sensory impressions. His view clearly contradicts representationalism, which Husserl fervently rejected (Box 3). According to contemporary versions of representationalism in the philosophy of mind, phenomenal qualia—specifically, the subjective “what it is like” aspect of our memory experiences—are part of its epistemic content (Shim, 2011; Liu, 2024a,b; Perrotta, 2025).

Box 3 **Phenomenology as the philosophy of direct experiences.**

Intentionality has its roots in phenomenology, a philosophical movement launched in the early 20th century by Edmund Husserl, a philosopher with a mathematical background. Husserl was interested in developing a general theory of inferential systems, whose basic tenet was that any phenomenological description proper is to be performed from a first-person point of view, to ensure that the respective object or event is described exactly as is experienced, or intended, by the subject. Husserl rejected “representationalist” accounts of intentionality, ascribing intentional experiences as intra-mental pictorial representations of objects, which could only yield a distorted picture of our phenomenal experience. He was interested in a form of reduction that goes back to the “things in themselves” as they appear to us, or to stand for things, properties, and states of affairs. Importantly, phenomenology, perhaps more than any other single movement in philosophy, has been key in bringing subjectivity and emotions to the foreground of philosophical consideration (Elpidorou and Freeman, 2014; Shim, 2011; Liu, 2024a).

In our model of memory representations, explicit memory provides a framework, grounded in both neuroscience and cognitive psychology, for defining subjective quality (see section 5). This proposal is confirmed by influential studies of episodic elements of explicit memory, corroborating the idea that qualia are reflections of the subjective “fingerprints” of the brain situated in transient neural networks activated in episodic memories (Edelman et al., 2011; Ward and Guevara, 2022). In addition, as previously argued, emotions contribute to creating a highly subjective “affectively colored” content of episodic memory through interactions between the amygdala and the hippocampus.

Taken together, the arguments listed here justify the conclusion that although qualia may subjectively appear to be irreducible qualities of experience, they can also be understood to arise from neurocognitive mechanisms of memory without any form of reduction, and while preserving the richness of our memory representations as described in our theoretical model.

Given these considerations, we further conclude that there is no need to assume a separate ontology for qualia and subjective experiences, as long as they can be conceptualized within the framework of cognitive neuroscience theories (Feinberg and Mallatt, 2019; Fazelpour and Thompson, 2015). Such a theory is not reductionistic but rather materialistic, proposing that memory representations are rooted in neural assemblies that give rise to conscious experiences.

## 7 Summary and conclusions

A substantial part of this article was devoted to revisiting the issue that occupied center stage in the philosophy of mind: the nature of human consciousness as it emerges from the brain, separating it from metaphysical and purely phenomenological connotations. This entailed the subsequent question of how human subjectivity emerges from the physical substrate of the brain, marking related issues such as intentionality, emergence, and qualia, the clefts between thoughts and feelings (see Figure 1 for the general outline of our review, and Box 1 for the concepts discussed in this paper.

An important platform in our discussion of philosophical concepts of the mind was the theory of multiple memory systems, which, in many respects, provided us with a neurocognitive framework for situating or reinterpreting the above issues. Although the general emphasis in this paper was on explicit forms of memory representations and the brain structures and pathways that give rise to conscious experience, a complete theory of consciousness should also incorporate implicit forms of memory that are not directly associated with conscious experiences. We further emphasize that interactions between explicit and implicit forms of memory are a crucial element of tasks performed in daily life, as well as of complex skills that integrate perceptual, motor, and feedback components of ongoing performance.

### 7.1 Bridging the gap between philosophy and neuroscience: reframing concepts from philosophy of mind

Box 4 summarizes the principal conclusions derived from our theory of memory representations, which we shall here briefly recapitulate. We propose that consciousness can be reframed in terms of access and phenomenal consciousness, referring, respectively, to the encoded and retrieved aspects of representations in explicit memory. In this context, consciousness is defined as the subjective experience of retrieved episodic elements of explicit memory, also referred to as phenomenal consciousness.

Box 4 **Major conclusions.**

• Our model of representationalism differentiates between explicit and implicit forms of multimodal memory networks, utilizing specific core structures and subcortical-cortical pathways.
• Consciousness can manifest itself in a variety of states, such as wakefulness, orienting, and voluntary attention.
• Phenomenal experience refers to the subjective experience of retrieved episodic elements of explicit memory, also known as phenomenal consciousness.
• The strong link between intentionality and mental representations allows us to redefine and expand intentionality through neuro-cognitive representations, capturing both the 'what is it' and 'what it is like' aspects in philosophy of mind.
• Emergence applies to a bottom-up process where individual neurons form small networks, which are then combined into larger unimodal and multimodal networks that create fully conscious, spatially integrated representations of objects (Pennartz, 2022).
• Phenomenal experiences (“qualia”) are anchored in episodic memory, which carries a personal signature and an emotional tone.
• Subjective experiences are intrinsic to our brain's neural circuits, emerging from interactions between emotion and memory structures. Thus, there is no need for a separate ontology of qualia and subjective experiences as long as they are conceptualized within neuroscience frameworks (Feinberg and Mallatt, 2019).

Regarding intentionality, this article argues that it is a fundamental element of conscious experience, residing in neural assemblies that incorporate memory representations. Representations harness the capacity of neural assemblies to predict or interpret events in the external world, utilizing the built-in modeling capacity of memory representations to transform physical reality into subjective experience. These views clearly confirm strong links with current definitions of phenomenology, emphasizing that every act of consciousness we perform, every experience we have, is intentional, i.e., essentially “consciousness” (Fahrenfort and Lamme, 2012).

From a similar perspective, emergence does not imply irreducibility. Instead, it can be conceived as a process realized in a bottom-up fashion by successive levels of single neurons forming small local networks, which are combined at subsequent levels into unimodal and multimodal multilevel networks, thereby creating (“emerging”) conscious representations of objects integrated in space (Pennartz, 2022).

According to our model of memory representations, explicit memory also provides a framework for defining subjective quality, as supported by studies of episodic memory in the human brain. These studies corroborate the idea that qualia reflect the subjective “fingerprints” of the brain, located in transient neural networks activated during the retrieval of episodic memories (Box 5).

Box 5 **Demystifying subjectivity.**

It may be only human to think of the mind as a mystery, just as with the immensity of the universe. This holds in particular for philosophers who support the idea of the ‘hard problem', arguing that no mechanistic or behavioural explanation could explain the character of a subjective experience, not even in principle. This view clearly draws on a strict version of phenomenology, the philosophical study of subjective, conscious experiences that aims to describe phenomena as they appear to the subject introspectively (Chalmers, 1996, 2005). Studies of the brain, in contrast, have adopted a broader definition of phenomenology, namely that phenomenal experiences are genuine in a material but non-reductionistic sense, reflecting transient activity in neural networks activated during episodic memories. Reciprocal connections between limbic and cortical regions enable the ‘colouring' of the content of episodic memory representations, as well as to interpret via introspection the related neural signals from a first-person affective perspective.

Subjective experiences are intrinsic to our brain's neural circuits, emerging from interactions between emotion and cognition. In sum, phenomenal experiences (“qualia”) are anchored in episodic memory networks that possess a personal signature and affective flavor. In conclusion, there seems to be no need for qualia as a separate ontological category to produce a state of subjective consciousness.

### 7.2 Evolution of representations

In his book Darwin's Dangerous Idea, Dennett developed the idea of a Darwinian process, “involving variation, selection and retention, as a generic algorithm that is substrate-neutral and could be applied to many fields of knowledge outside of biology” (Dennett, 1995). Similarly, Bering and Schackelford proposed that several Darwinian selection principles could be grounded in representational systems, in which conscious motives have inserted themselves at the level of the gene, fundamentally changing the nature of hominid evolution (Bering and Schackelford, 2004).

A powerful concept directly relevant to a theory of memory representation is predictive coding, also known as predictive processing. This theory posits that an internal model, formed through learning and stored in long-term memory, serves as a model or template in the brain to compare input with existing representations. Predictive coding could also take the form of more complex predictive abilities, such as planning, anticipating future events, and imagination. Although the theory suggests that these functions rely on highly complex cognitive processes, they could also have “emerged gradually during evolution from simpler predictive and error correction loops in the brains of our earlier evolutionary ancestors” (quote from Pezzulo et al., 2021). A related hypothesis concerns the evolution of language abilities in the human species. These could have evolved from more basic human abilities to recognize and remember sequences, a crucial evolutionary step toward human language and a key trait in the development of human culture and thought (Jon-And et al., 2023).

Indeed, the dense distributed networks of our brain that underlie the functional operational space of our memory system could have evolved in such a way as to optimize adaptations to a world where symbolic communication became equally important as the direct need to survive in a physical sense (Donald, 1991; Buckner and Krienen, 2013). Symbolic reference systems are intrinsic to the brain's neural architecture and gradually became grounded in neural representations of our declarative memory system (Deacon, 1997). A larger brain enabled the human brain to evolve into a unique organ, distinct from those of other mammals. Manifested not only in a larger volume relative to body size, but also in specific adaptations of its connectome (Ardesch et al., 2019). This likely involved a larger volume of association areas than of primary areas, as well as stronger connectivity of long-range reciprocal fibers in the dorsal and ventral routes of the brain. Together, these neural architectures would enable easier access to the areas involved in attentional control and consciousness, as well as the area involved in language processing.

## Footnotes

1 The present definitions of representations come close to that of enactivism: the theory that perception creatively matches its actions to the requirements of its environment. Enactivism encompasses representations of action and object schemas, which are assumed to be processed in sensory, motor, and multimodal areas of the brain.

2 These considerations clearly refer to the problem of “free will”, a person's perception that thoughts and movements made are deliberate conscious choices. Free will is extensively discussed in the literature (Hallet, 2007, 2016; Lavazza and Inglese, 2015), but it is too complex a subject to be addressed in the already wide range of issues discussed in the present article.

3 In a different context, the term “state” is used to indicate the global state of consciousness, varying between sleep, drowsiness, and wakefulness, occurring with a much longer-lasting cycle, dominated by the circadian rhythm.

4 Notice that encoding or consolidation applies to the initial phase of learning, followed by more permanent structural changes designated as storage. Retrieval refers to the process of utilizing stored information to create a conscious representation or to execute learned behavior, such as a motor act.

5 Theories of automaticity (outside the field of the present article) propose that after sufficient practice, controlled effortful processing transits to effortless long-term memory detection (Atkinson and Shiffrin, 1968; Fisk and Schneider, 1984; Strayer and Kramer, 1990; Schneider and Chein, 2003; Chein and Schneider, 2012). This implies that the retrieval of content of explicit memory representations may occur automatically, without any conscious awareness.

6 The current definition of PC as the process of retrieving encoded content to create conscious experience aligns well with the properties of the P3 (P300) component of the brain's event-related potential (ERP), which reflects a conscious “match” of a stimulus with a stored memory in long-term memory (Polich and Kok, 1995; Kok, 2001; Donchin and Coles, 2010).

7 Following the evidence discussed earlier, we assume that the two subcategories of explicit memory, episodic and semantic memory, rely on identical subcortical-cortical circuits. This, however, does not preclude differentiating between various subforms of explicit memory representations based on their specific category, episodic content, or complexity at the cortical level.

8 A meta-analysis of the effects of extensive practice of tasks in the domains of skill learning as well as declarative learning indicated: (a) generalized reductions in regional activity by reducing the load on the cognitive control mechanisms, (b) that these control mechanisms are domain-general, and (c) that no separate processing pathway emerges as skill develops (Chein and Schneider, 2012).

9 A principle underlying the long-ranging connections with the cerebral cortex associated with conscious perception is re-entrant pathways, a term introduced by Gerald Edelman (Edelman, 1992; Edelman et al., 2011; Edelman and Gally, 2013). Re-entry is also referred to as recurrent, recursive, or top-down-bottom-up processing. It is the ongoing bidirectional exchange of signals along reciprocal long-distance axonal fibers, linking two or more functionally segregated brain areas (Dijkstra et al., 2017; Gilbert and Li, 2013). In the brain, these linkages are manifested as either some form of interaction or temporal coherence among distributed cortical areas, without the intervention of a higher coordination mechanism (Milner, 1974; Singer, 2001; Dehaene et al., 2003).

10 Predictive coding is related to Bayesian inference. Predictive coding, however, specifies that prediction errors, rather than inputs, should be represented, while remaining agnostic about how predictions are made. In contrast, Bayesian inference provides an optimal calculus for computing predictions, but does not specify the underlying neural representation (Aitchison and Lengyel, 2017).

11 The Feature Integration theory of Treisman (Treisman and Gelade, 1980) is another example of a theory of conscious awareness based on the principle of matching sensory input with a stored memory representation. The theory states that voluntary spatial attention functions as a searchlight directed to the target's location in a location map, while “binding” different features of the target stimulus into a perception of the whole object, which is then matched with a predefined representation in memory.

12 Defined as: “the simplified, user-friendly interface that the brain creates to interact with the complex reality of its own internal workings” (Dennett, 1991, p. 309–314).
